# Toward the benefit and value of immune treatment beyond progression in lung cancer? Insights from a systematic review and meta-analysis

**DOI:** 10.3389/fimmu.2025.1547978

**Published:** 2025-04-01

**Authors:** Rui Huangfu, Kun Hou, Jie Zhao, Shikui Wu, Yaodong Ping

**Affiliations:** ^1^ School of Pharmacy, Inner Mongolia Medical University, Hohhot, China; ^2^ Department of Pharmacy, Peking University Cancer Hospital Inner Mongolia Hospital, Hohhot, China; ^3^ Department of Pharmacy, Key laboratory of Carcinogenesis and Translational Research (Ministry of Education/Beijing), Peking University Cancer Hospital and Institute, Beijing, China

**Keywords:** immune treatment beyond progression, lung cancer, effectiveness, safety, beneficiary population

## Abstract

**Objective:**

Immune treatment beyond progression (ITBP) has emerged as a novel therapeutic strategy in oncology. This systematic review and meta-analysis aim to evaluate the efficacy and safety of ITBP in patients with lung cancer, while also identifying characteristics of populations that may benefit most from this treatment approach.

**Methods:**

This study adheres to the PRISMA guidelines. We searched PubMed, Embase, and the Cochrane Library for relevant literature on immunotherapy for lung cancer, using self-constructed databases up until February 1, 2024. The study includes real-world data from patients with lung cancer undergoing ITBP, categorized into two groups: non-ITBP (NTBP) and ITBP. Two authors independently conducted literature screening, quality assessment, and data extraction. The primary efficacy indicators include overall survival (OS), progression-free survival (PFS), objective response rate (ORR), and disease control rate (DCR). The safety indicator assessed was the incidence of immune-related adverse events (irAEs).

**Results:**

We included 9 studies with a total of 5,141 patients with lung cancer, comprising 2,051 patients in the ITBP group and 3,090 in the NTBP group. Patients receiving ITBP showed significantly better outcomes than those receiving NTBP, including superior OS and PFS following treatment beyond progression (OS: hazard ratio (HR) 0.72, 95% confidence interval (CI) 0.68-0.77, P < 0.05; PFS: HR 0.63, 95% CI 0.51-0.78, P < 0.05). Additionally, the ITBP group demonstrated higher ORR and DCR (ORR: odds ratio (OR) 0.48, 95% CI 0.31-0.75, P < 0.05; DCR: OR 0.37, 95% CI 0.24-0.57, P < 0.05). No significant difference in the incidence of irAEs was found between the two groups (OR 1.24, 95% CI 0.83-1.85, P > 0.05). Subgroup analysis revealed that factors such as age, gender, lung cancer subtype, and smoking history significantly influenced OS outcomes in the ITBP group.

**Conclusion:**

Our findings suggest that ITBP is an effective treatment strategy for patients with lung cancer. Further research should focus on identifying specific patient populations that benefit from ITBP and exploring the potential efficacy of combining ITBP with other therapeutic regimens.

**Systematic Review Registration:**

https://www.crd.york.ac.uk/prospero/, identifier CRD42024513475.

## 1 Introduction

Primary bronchogenic lung cancer, commonly referred to as lung cancer, is one of the most prevalent and deadly cancers worldwide, including in China (1). In 2022, lung cancer was the leading cause of new cases and deaths from malignant tumors in China (2). Lung cancer is divided into small-cell (SCLC) and non–small-cell (NSCLC) lung cancer, with NSCLC accounting for more than 85% of cases. Early-stage lung cancer typically presents with no obvious symptoms, and most patients are diagnosed at advanced stages, resulting in a 5-year survival rate of approximately 20% for those with advanced disease (3). Therefore, identifying effective treatment strategies is critical.

In recent years, immunotherapy has demonstrated considerable promise in the treatment of lung cancer. Unlike conventional therapies that target the tumor cells themselves, immunotherapy focuses on overcoming immune suppression within the tumor microenvironment, thereby activating the immune system to combat cancer cells (4). The use of immune checkpoint inhibitors (ICIs) has opened up new therapeutic avenues for patients with lung cancer. Notable clinical trials such as KEYNOTE 024 (5) and IMpower150 (6) have shown that PD-1/PD-L1 inhibitors, whether used alone or in combination therapies, can be effective as first-line treatments for lung cancer, offering substantial clinical benefits. Moreover, while the five-year overall survival (OS) rate with chemotherapy is approximately 10%, immunotherapy has yielded a 5-year OS ranging from 13% to 31.9%. Despite these long-term survival benefits, many patients eventually develop drug resistance, with primary resistance occurring in 7% to 27% of those undergoing first-line immunotherapy, and this rate rises to between 20% and 44% for second-line treatments (7).

Immunotherapy has been shown to alter the biological characteristics of tumors, allowing for sustained survival benefits even after imaging progression, a phenomenon referred to as post-progression prolongation of survival (PPPS). Although some patients exhibit disease progression in imaging, clinical symptoms may improve, likely due to enduring antitumor immune responses, including continuous recognition and memory of tumor antigens. As cycles of antitumor immunity repeat, the immune response may strengthen, offering potential long-term benefits. This suggests that continuing immune treatment beyond progression (ITBP) could be a promising strategy in cancer treatment.

In oncology, ITBP refers to continuing immunotherapy, with either the same or different ICIs, after disease progression during previous ICI treatment. This approach aims to maintain immunotherapy despite tumor progression, tailored to each patient’s condition. In cases of slow progression or pseudo-progression, continuing ITBP could be beneficial, whereas it is not recommended for patients whose condition deteriorates after progression. Secondary resistance could be addressed by combining ITBP with oncolytic viruses or anti-angiogenic therapies, potentially improving immune resistance and reactivating immune function (8).

Initial studies such as KEYNOTE 010 (9) demonstrated that patients who experienced disease progression after 35 cycles of pembrolizumab treatment still benefited from further pembrolizumab therapy, with an ORR of 35% and a DCR of 85%. Furthermore, these patients achieved a 5-year OS rate of approximately 85%. In a recent Phase III study conducted by Professor Gandara’s team in the United States (the OAK study), patients who continued atezolizumab after progression had a median OS of 12.7 months, compared with 8.8 months in those who switched to docetaxel (NTBP). This suggests that continued treatment with atezolizumab post-progression is associated with better outcomes and superior tolerability than docetaxel (10). A retrospective study from Peking Union Medical College Hospital confirmed that continuing immunotherapy post-progression significantly extended OS, even in patients with stable tumor mutation burden (TMB) (11). However, contrasting results were reported in a cohort study from Japan, which included 15 patients with NSCLC who received PD-1 inhibitors, as crossover therapy after first-line PD-L1 inhibitor treatment did not demonstrate significant survival benefits. The divergent findings between these two studies may be attributed to differences in patient characteristics, particularly because the Japanese study included only patients with low PD-L1 expression (PD-L1 < 50%), which may have contributed to the suboptimal efficacy of ITBP (12). A real-world study conducted by Stinchcombe (13) involving 4,223 patients further substantiated the efficacy of ITBP, showing better survival outcomes in patients receiving immunotherapy beyond progression (OS: ITBP vs NTBP, 11.5 vs 5.1 months, HR 0.69, P < 0.001).

Despite promising results from various studies, the clinical application of ITBP for lung cancer remains inconsistent, with few high-quality evidence-based guidelines. Consequently, clinical decision-making often relies on physicians’ experiences. This study aims to contribute to the growing body of evidence by including real-world data from patients with lung cancer undergoing ITBP and conducting a systematic review and meta-analysis to assess its efficacy and safety. It also seeks to explore the characteristics of populations that may benefit from ITBP, providing crucial support for its clinical application.

## 2 Materials and methods

This systematic review and meta-analysis were conducted in accordance with the PRISMA guidelines (PRISMA checklist available in **Supplementary Material 1**). The study protocol was registered with the International Prospective Register of Systematic Reviews (PROSPERO) database (CRD42024513475).

### 2.1 Eligibility criteria

We included real-world studies (RWS) on ITBP for lung cancer. RWS gathers clinical data from patients treated under actual conditions in clinical practice, offering insights into the efficacy and safety of treatments, drugs, and medical technologies. Unlike randomized controlled trials (RCTs), which enforce strict experimental conditions, RWS captures patient outcomes in natural clinical settings, where patient selection and treatment approaches vary. RWS commonly encompasses a variety of study designs, including observational cohort studies, case-control studies, cross-sectional studies, and retrospective database analyses. Common data sources for RWS include electronic health records (EHRs), medical insurance databases, patient registries, clinical practice observations, and patient-reported outcomes. The studies included in our analysis specifically focused on continuing immunotherapy in patients with lung cancer who had experienced progression as per the Response Evaluation Criteria in Solid Tumors (RECIST) version 1.1 (14).

### 2.2 Search strategy

We conducted a search of electronic databases including PubMed, Embase, Web of Science, and the Cochrane Central Register of Controlled Trials (CENTRAL) for relevant studies from inception to February 1, 2024. The search strategy combined the following keywords: (“lung cancer” OR “non-small-cell lung cancer” OR “small-cell lung cancer”) AND (“immune checkpoint inhibitors” OR “PD-1 inhibitors” OR “PD-L1 inhibitors” OR “CTLA-4 inhibitors”) AND (“cross-line therapy” OR “treatment beyond progression” OR “retreatment”) to search the titles and abstracts of queried literature (**Supplementary Material 2**). We also manually reviewed the reference lists of previous systematic reviews and included studies.

### 2.3 Criteria for inclusion and exclusion

Inclusion criteria encompassed RWS with the following characteristics: (I) Patients aged ≥18 years with pathologically confirmed lung cancer who had previously undergone immunotherapy and discontinued treatment due to progression (PD); (II) Intervention: experimental group (ITBP group) – following PD during first-line ICI treatment, patients continued with the same or different ICIs in subsequent lines, either as monotherapy or in combination with other regimens; control group (NTBP group) – after PD during first-line ICI therapy, patients either switched to nonimmunotherapy treatment or discontinued treatment; (III) Outcomes: at least one of the following: OS, PFS, ORR, DCR, or immune-related adverse events (irAEs). OS was defined as the time from ITBP initiation to death from any cause; PFS as the time from ITBP initiation to PD; ORR as the proportion of patients achieving complete response (CR) or partial response (PR); DCR as the proportion of patients achieving CR, PR, or stable disease (SD). All irAEs were recorded and graded according to the Common Terminology Criteria for Adverse Events (CTCAE), with severity classified into grades 1 through 5. The exclusion criteria were as follows: (I) Incomplete data or inability to obtain the full text; (II) Duplicated publications; (III) Systematic reviews, case reports, reviews, letters, meeting abstracts, comments, or unpublished data.

### 2.4 Data extraction

Articles retrieved from the databases were initially screened using EBM AI-Reviewer, an artificial intelligence tool for literature screening based on the PICOS (population, intervention, comparison, outcomes, studies) framework. Two authors (HFR and HK) independently assessed the eligibility of all studies based on the aforementioned inclusion and exclusion criteria after reviewing the study title, abstract, and full text in succession. Studies were included in only the systematic review (but not the meta-analysis) if their findings were relevant to the research question, but data were not available for quantitative analysis. Any disagreement among authors was discussed and reconciled by the corresponding author (PYD). Two authors (HFR and HK) independently extracted data and assessed study quality, resolving discrepancies through discussion and submitting unresolved issues to a third researcher for final resolution. Extracted variables included: (1) General information: title, author, research type, publication year, and source; (2) Study characteristics: patient tumor type, initial immunotherapy drugs, therapy types, use of combination therapy, study sample size, and outcome measures.

### 2.5 Quality evaluation and bias risk assessment

Two independent evaluators assessed the quality of included studies using the Quality Assessment Tool for Systematic Reviews of Observational Studies - Real World Studies (QATSM-RWS). This tool, designed for systematic reviews of observational studies such as cohort and case-control studies, contains five modules: introduction, methods, results, discussion, and additional considerations. Each module includes several sub-items, totaling 14 items. Responses were scored as “yes” (1 point), “no” (0 points), or “unclear” (0.5 points), with a maximum score of 14. In cases of disagreement, consensus was reached through discussion or consultation with a third researcher (15).

### 2.6 Statistical analyses

Hazard ratio (HR) was selected as the effect size for PFS and OS, while odds ratio (OR) was used for ORR and DCR. We conducted meta-analysis of effect sizes using Review Manager 5.4.1 software, with statistical significance set at P < 0.05. Heterogeneity was assessed using the I² test, with values combined to gauge the magnitude of heterogeneity. A fixed-effects model was employed for all meta-analyses. If P > 0.1 and I² ≤ 50%, heterogeneity was considered low; if P ≤ 0.1 and I² > 50%, it indicated high heterogeneity (16). Sensitivity analysis was performed using STATA version 16.1 to test the robustness of the results. Publication bias was analyzed using Begg’s and Egger’s tests, with P < 0.05 indicating significant publication bias.

## 3 Results

### 3.1 Literature search results

**Figure 1:**
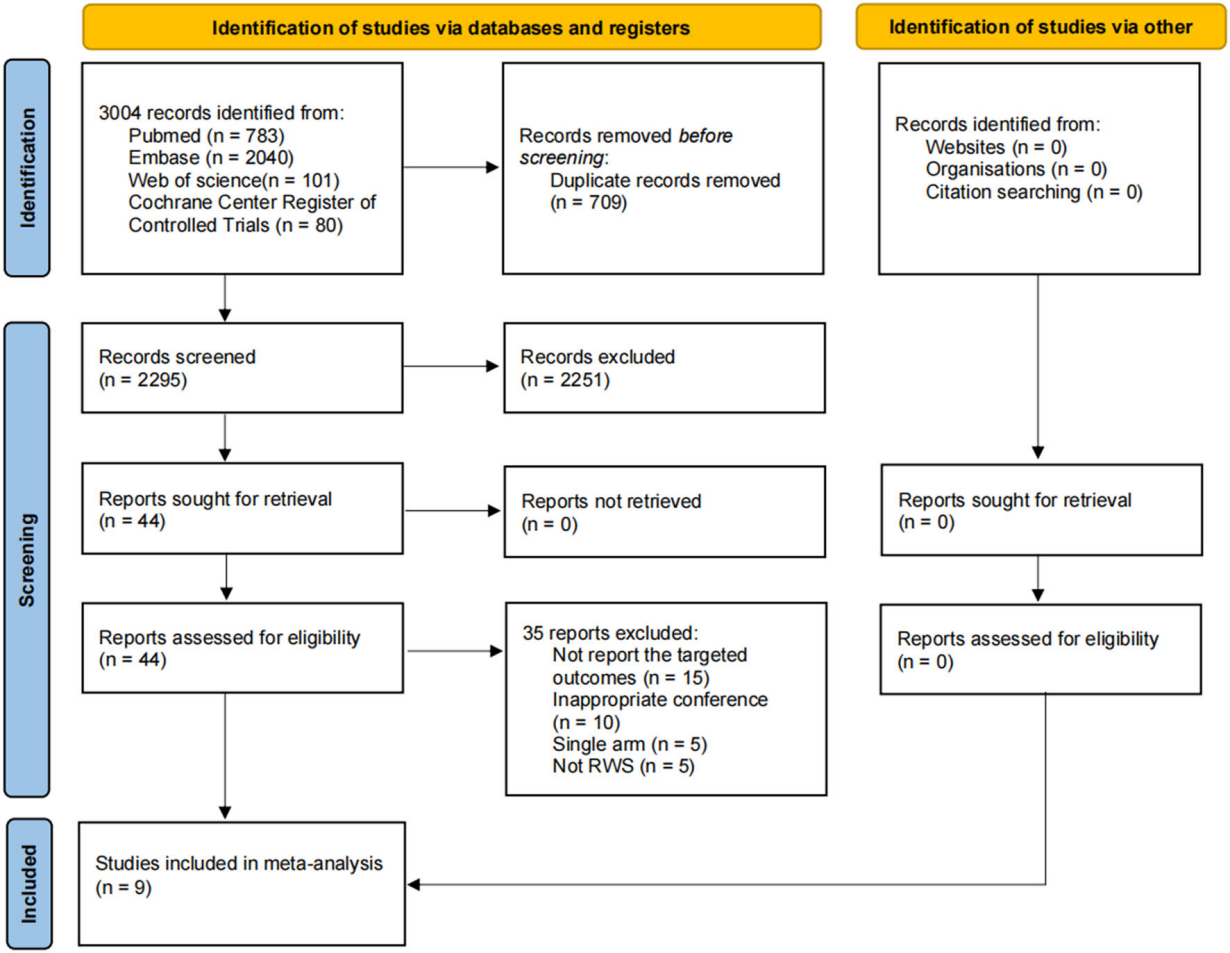
PRISMA flowchart for selection of relevant studies in this meta-analysis.

A total of 3,004 candidate references were identified through electronic database searches, with no additional references found via manual search. After removing 709 duplicates, 2,295 references were excluded following a thorough review of titles and abstracts. Ultimately, 44 references were deemed relevant and underwent full-text review. Of these, 15 studies did not report the targeted outcomes, 10 were from inappropriate conferences, 5 were single-arm studies, and 5 did not meet the criteria for RWS. Based on the inclusion and exclusion criteria, 9 studies were included in this systematic review and meta-analysis (**Figure 1**).

### 3.2 Characteristics of included studies

The nine included studies were all real-world, retrospective, observational studies, eight of which focused on NSCLC and one on SCLC. These studies included 5,141 patients, of whom 2,051 (40.0%) were in the ITBP group and 3,090 (60.0%) were in the NTBP group. Nine studies provided OS data, four provided PFS data, five provided ORR and DCR data, and three provided irAEs (**Table 1**).

**Table 1:** Baseline characteristics and primary results of included studies.

Study, years	Region	Study design	Number (T/C)	Median Age (T)	Gender (T) (%)		TNM staging (T)	Histology (T) (%)		Smoking history (T) (%)	Gene mutation status (T)		ECOG- PS≥2 (T) (%)	Lines of being with ITBP	Initial ICIs	ITBP	NTBP	Outcomes
					Male	Female		SCC	Non- SCC		EGFR	ALK						
Thomas E. Stinchcombe, 2020 (13)	Worldwide	Retrospective observational	1668/ 2555	69	882 (52.9)	786 (47.1)	I-IV	452 (27.1)	1164 (69.8)	1477(86.8)	62(5.3)	7(0.6)	236(14.1)	≥2	ICIs M/ ICIs CT	ICIs M/ ICIs CT	Discontinue ICIs	①
Xiangwei Ge, 2020 (17)	China	Retrospective observational	39/86	56	29 (74.4)	10 (25.6)	IIIB/IV	17 (43.6)	22 (56.4)	23(59.0)	6(15.4)	2(5.1)	3(7.7)	≥2	ICIs M/ ICIs CT	ICIs M/ ICIs CT	Discontinue ICIs	①②③④
Yixing Wang, 2023 (18)	China	Retrospective observational	33/26	60	30 (90.9)	3 (9.1)	IIIC/IV	13 (39.4)	20 (60.6)	20(60.6)	NA	NA	3(9.1)	2	PD-1 CT	PsC + ICIs	PsC	①②③④⑤
Yuanyuan Cheng, 2023 (19)	China	Retrospective observational	93/76	NA	82 (88.2)	11 (11.8)	NA	54 (58.1)	39 (41.9)	59(63.4)	0	0	10(10.8)	2	ICIs CT	ICIs CT	Discontinue ICIs	①③④
Giulio Metro, 2019 (20)	Worldwide	Retrospective observational	18/42	71	11 (61.1)	7 (38.9)	NA	6 (33.3)	8 (50.0)	15(83.3)	0	0	6(33.3)	2	Pembro	Pembro	Chemo	①②③④⑤
Biagio Ricciuti, 2019 (21)	Italy	Retrospective observational	60/116	62	38 (63.3)	22 (36.7)	NA	9 (15.0)	50 (83.3)	54(90.0)	6(10.0)	0	14(23.3)	≥2	Nivo	Nivo	Discontinue ICIs	①⑤
Takatoshi Enomoto, 2022 (22)	Worldwide	Retrospective observational	28/67	73	16 (57.1)	12 (42.9)	NA	12 (42.9)	16 (57.1)	22(78.6)	1(3.6)	NA	6(21.4)	3	Nivo	Nivo	Other treatment	①
Sang Eun Won, 2019 (23)	Korea	Retrospective observational	67/67	64	57 (85.1)	10 (14.9)	NA	NA	NA	NA	NA	NA	9(13.4)	3	Nivo, Atezo, Pembro, Avel, Durva	ICIs	Discontinue ICIs	①
Lingling Li, 2020 (24)	China	Retrospective observational	45/55	61	33 (73.3)	12 (26.7)	NA	NA	NA	30(66.7)	NA	NA	15(33.3)	≥2	ICIs M/ ICIs CT	ICIs CT	ICIs<6w	①②③④

Outcomes: ①OS; ②PFS; ③ORR; ④DCR; ⑤irAEs; Nivo, Nivolumab; Atezo, Atezolizumab; Pembro, Pembrolizumab; Avel, Avelumab; Durva, Durvalumab; ICIs, Immune Checkpoint Inhibitors; CT, combination therapy; M, monotherapy; PsC, patients received physician’s choice; Chemo, Chemotherapy; NA, Not Available; Lines of being with ITBP, The line number from which a patient begins with ITBP, if a patient uses immunotherapy as first-line therapy and continues to use immunotherapy as second-line therapy after PD, the “Lines of being ITBP” are 2 and so on.

### 3.3 Quality assessment

The methodological quality of the included studies was evaluated using the QATSM-RWS (**Table 2**). Among the nine studies, five received a score of 13.5 points, three received 13 points, and one received 11.5 points. These scores suggest that the overall methodological quality of the included studies was generally high, with most studies exhibiting robust design and reporting. Minor variations in scores reflect differences in methodological rigor, which were taken into account when interpreting the results.

**Table 2:** Quality assessment of included studies using the QATSM-RWS tool.

SN	Item	Thomas E. Stinchcombe	Xiangwei Ge	Yixing Wang	Yuanyuan Cheng	Giulio Metro	Biagio Ricciuti	Takatoshi Enomoto	Sang Eun Won	Lingling Li
Introduction										
1	Was (were) the research question/objective (s) of the study clearly defined?	1	1	1	1	1	1	1	1	1
2	Does the study explain the scientific background and rationale for the investigation being reported?	1	1	1	1	1	1	1	1	1
Methods										
3	Are the study sample demographic characteristics clearly described and defined?	1	1	1	1	1	1	1	1	1
4	Were the sources of data used for the study clearly described?	1	1	1	1	1	1	1	1	1
5	Are the study design and data analysis applied in the study described in enough detail?	0.5	1	1	1	1	1	1	1	1
6	Was the chosen sample size appropriate for the objective of the study?	1	1	1	1	1	1	1	1	1
7	Are the inclusion and exclusion criteria applied to the study described in enough detail?	0	1	0.5	1	1	0.5	0.5	1	1
8	Were the outcomes assessed in the study appropriate and clearly defined?	1	1	1	1	1	1	1	1	1
9	Was the follow-up of participants complete and long enough?	0.5	0.5	0.5	0.5	0.5	0.5	0.5	0.5	0.5
10	Were the methods of the study clearly described to enable them to be repeated?	0.5	1	1	1	1	1	1	1	1
Results										
11	Are the reported results clear and comprehensible?	1	1	1	1	1	1	1	1	1
Discussion										
12	Were the conclusions/recommendations of the study justified and based on the study results?	1	1	1	1	1	1	1	1	1
13	Was there a statement disclosing the potential conflict of interest of researcher(s)?	1	1	1	1	1	1	1	1	1
Others										
14	Was there a statement disclosing the source of funding for the study that may affect the authors’ interpretation of the results?	1	1	1	1	0	0	1	1	1
	**Total**	11.5	13.5	13	13.5	13.5	13	13	13.5	13.5

“yes” (1 point), “no” (0 points), or “unclear” (0.5 points).

### 3.4 Efficacy

#### 3.4.1 OS

**Figure 2:**
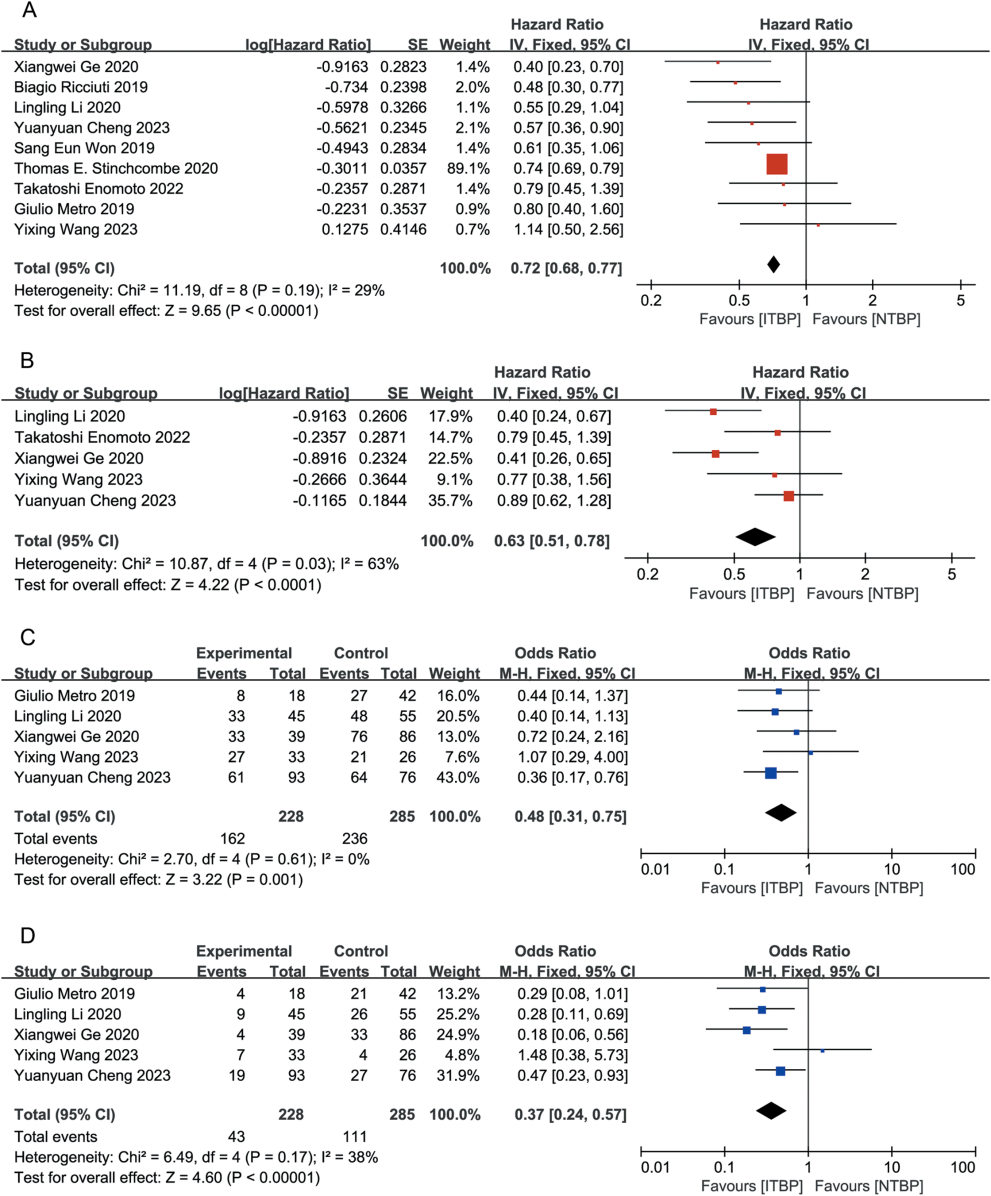
Forest plot of meta-analysis for the effects of ITBP on patients with lung cancer. **(A)** OS; **(B)** PFS; **(C)** ORR; **(D)** DCR.

All nine studies reported OS data, with an I² of 16%. Using a fixed-effects model, the HR for OS was 0.72 (95% CI 0.68-0.77, P < 0.05). This result suggests that the ITBP group significantly prolonged OS in patients with lung cancer compared with the NTBP group (**Figure 2A**).

#### 3.4.2 PFS

Five of the nine studies reported PFS outcomes, with an I² of 63%. The combined analysis using a fixed-effects model showed a HR of 0.63 (95% CI 0.51-0.78, P < 0.05), indicating that patients in the ITBP group had significantly prolonged PFS compared with those in the NTBP group (**Figure 2B**).

#### 3.4.3 ORR

Five studies reported ORR data, with an I² of 0%. The fixed-effects model revealed that the ITBP group had a higher ORR than the NTBP group, with a statistically significant difference (OR 0.48, 95% CI 0.31-0.75, P < 0.05) (**Figure 2C**).

#### 3.4.4 DCR

Five studies reported DCR data with an I² of 38%. The analysis showed that the ITBP group had a higher DCR than the NTBP group, with a statistically significant difference (OR 0.37, 95% CI 0.24-0.57, P < 0.05) (**Figure 2D**).

### 3.5 Subgroup analysis of OS and PFS

**Figure 3:**
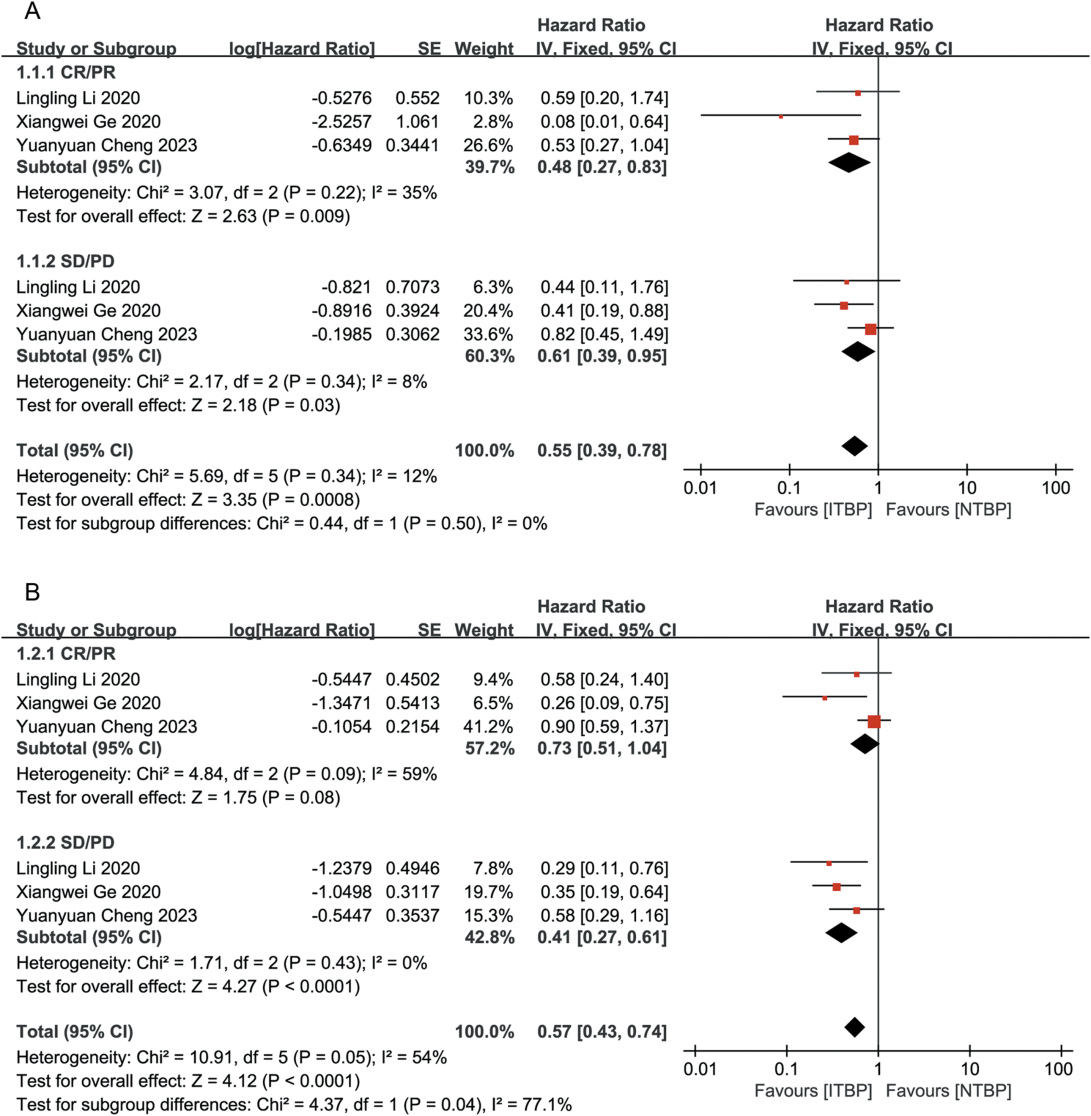
Subgroup analysis of OS **(A)** and PFS **(B)** by optimal response to initial immunotherapy.

Patients who achieved CR or PR had a greater reduction in the risk of death than those who achieved SD/PD (HR 0.48, 95% CI 0.27-0.83, P < 0.05 vs. HR 0.61, 95% CI 0.39-0.95, P < 0.05), with a statistically significant difference (P < 0.05) (**Figure 3A**). Patients who achieved CR/PR showed a risk reduction of 27%, but this difference was not statistically significant (HR 0.73, 95% CI 0.51-1.04, P > 0.05). In contrast, those who achieved SD/PD experienced a significant reduction in risk (HR 0.41, 95% CI 0.27-0.61, P < 0.05) (**Figure 3B**). These results suggest that OS and PFS were better in the ITBP group than in the NTBP group.

**Figure 4:**
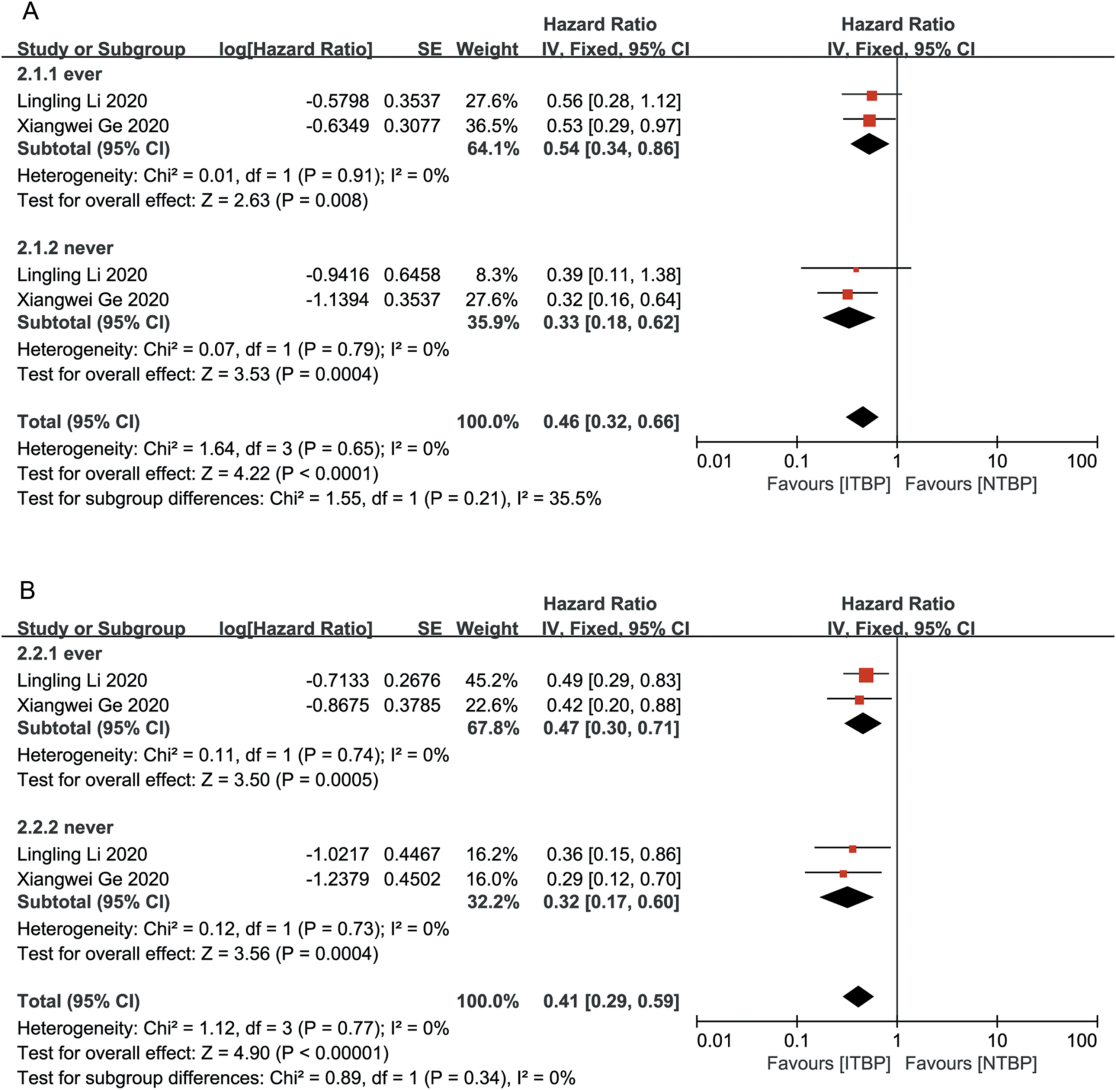
Subgroup analysis of OS **(A)** and PFS **(B)** with and without smoking history.

Patients who had never smoked showed significantly higher OS (HR 0.33, 95% CI 0.18-0.62) and PFS (HR 0.32, 95% CI 0.17-0.60) than those with a history of smoking, with statistically significant differences (P < 0.05) (**Figure 4**).

**Figure 5:**
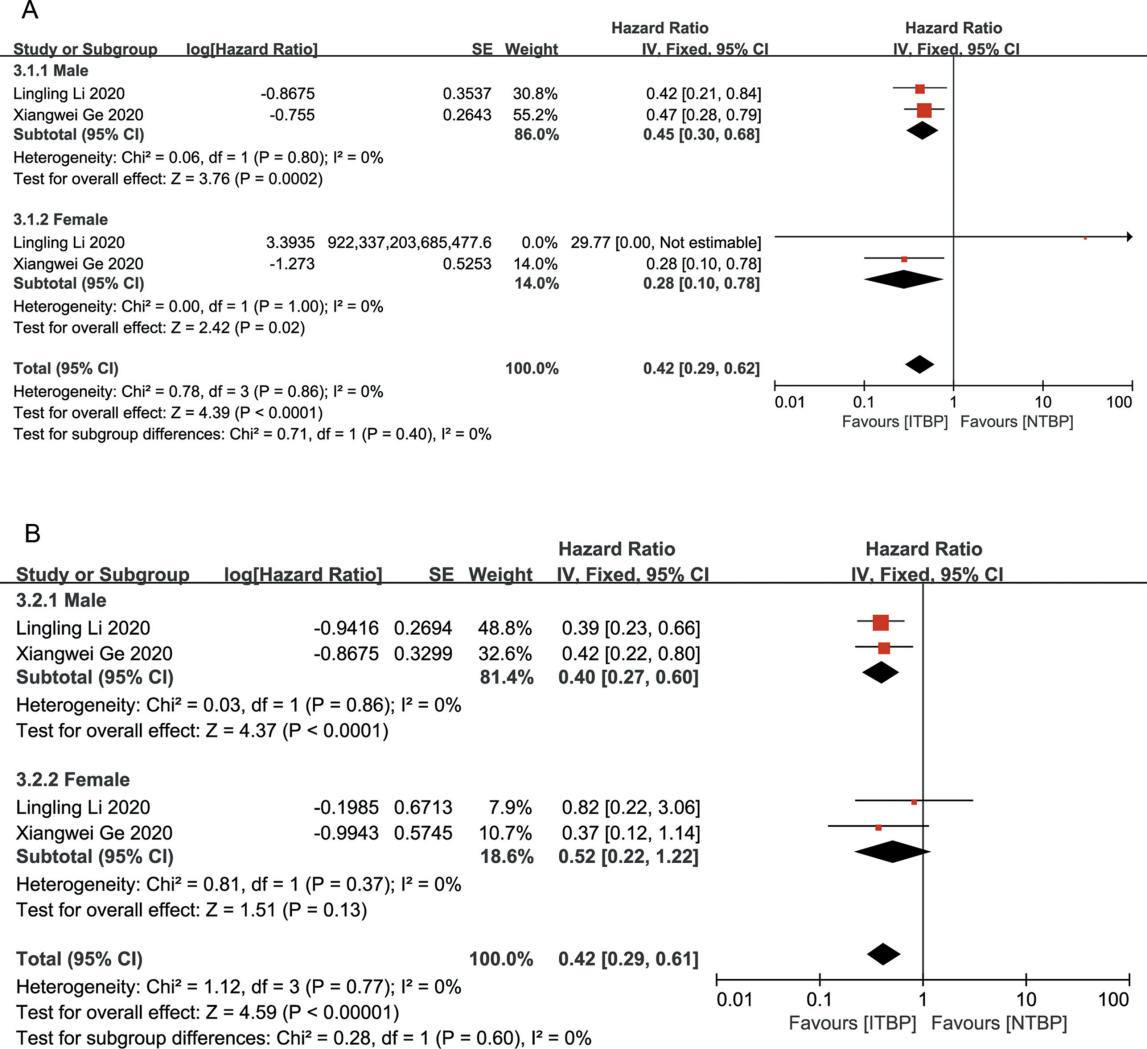
Subgroup analysis of OS **(A)** and PFS **(B)** by gender.

Both male and female patients in the ITBP group exhibited significant improvements in OS (HR 0.42, 95% CI 0.29-0.62, P < 0.05) and PFS (HR 0.42, 95% CI 0.29-0.61, P < 0.05), with no heterogeneity between genders (I² = 0%) (**Figure 5**).

**Figure 6:**
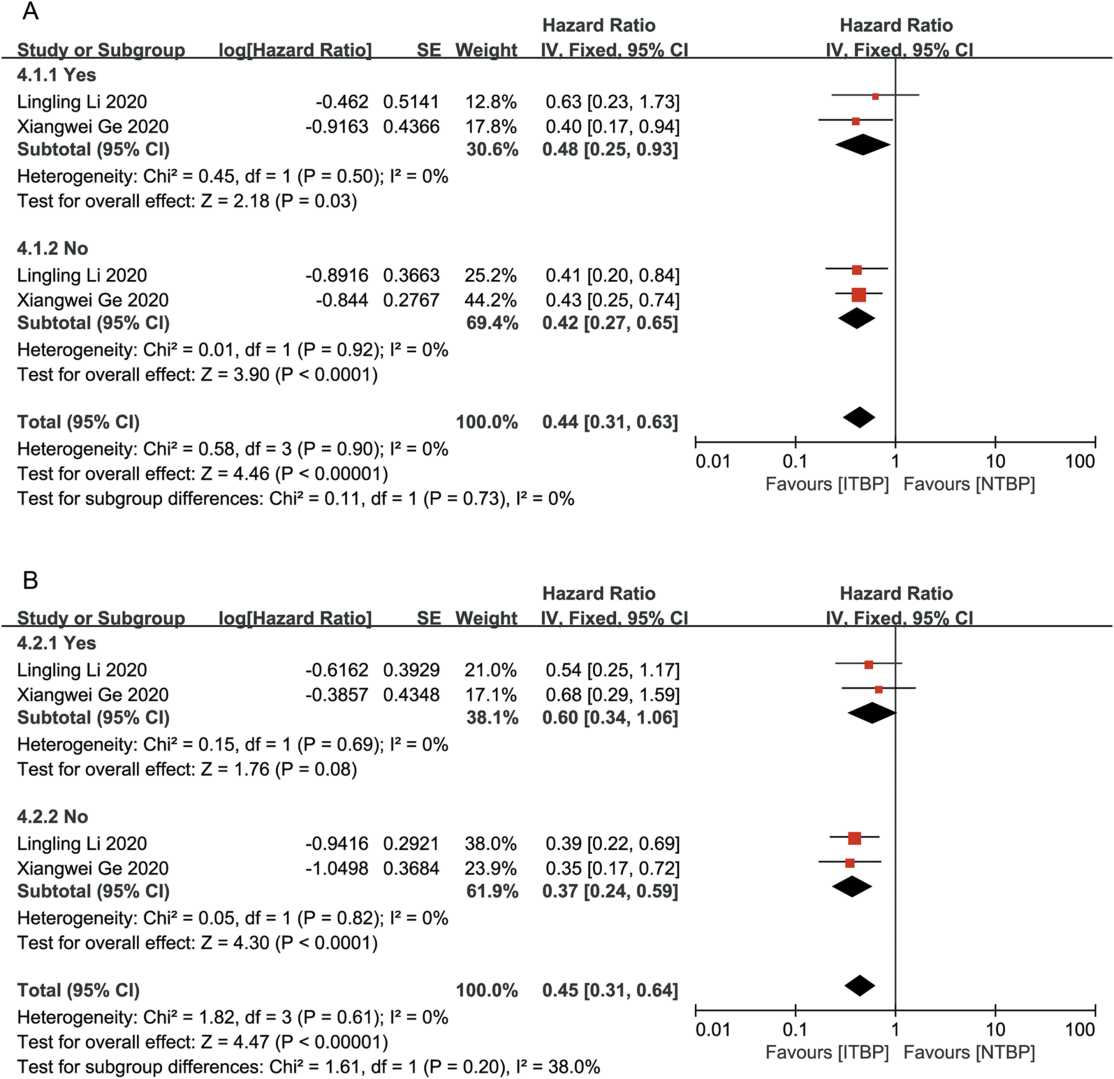
Subgroup analysis of OS **(A)** and PFS **(B)** for brain metastases.

**Figure 7:**
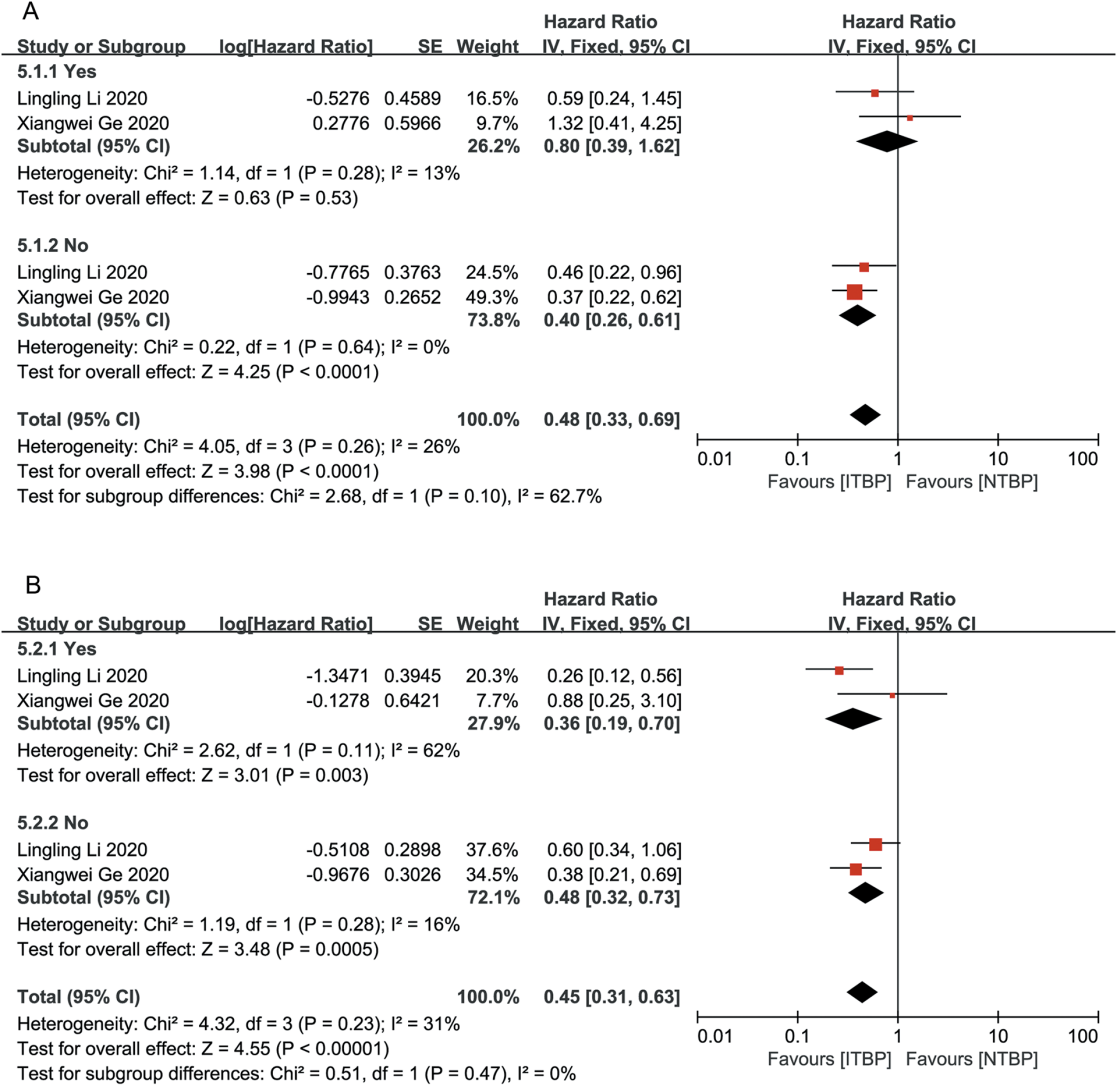
Subgroup analysis of OS **(A)** and PFS **(B)** for liver metastases.

Subgroup analysis based on the presence or absence of brain and liver metastases showed that patients without brain and liver metastases had significantly improved OS and PFS, with statistically significant differences (P < 0.05). ITBP in patients with brain metastases improved OS (HR 0.48, 95%CI 0.25-0.93, P < 0.05) but showed no significant difference in PFS (HR, 0.60; 95% CI: 0.34-1.06, P > 0.05); furthermore, ITBP in patients with liver metastases showed improved PFS (HR 0.36, 95% CI 0.19-0.70, P < 0.05) but showed no significant difference in OS (HR 0.80, 95% CI 0.39-1.62, P > 0.05) (**Figures 6**, **7**).

### 3.6 Sensitivity analyses and publication bias assessment

**Figure 8:**
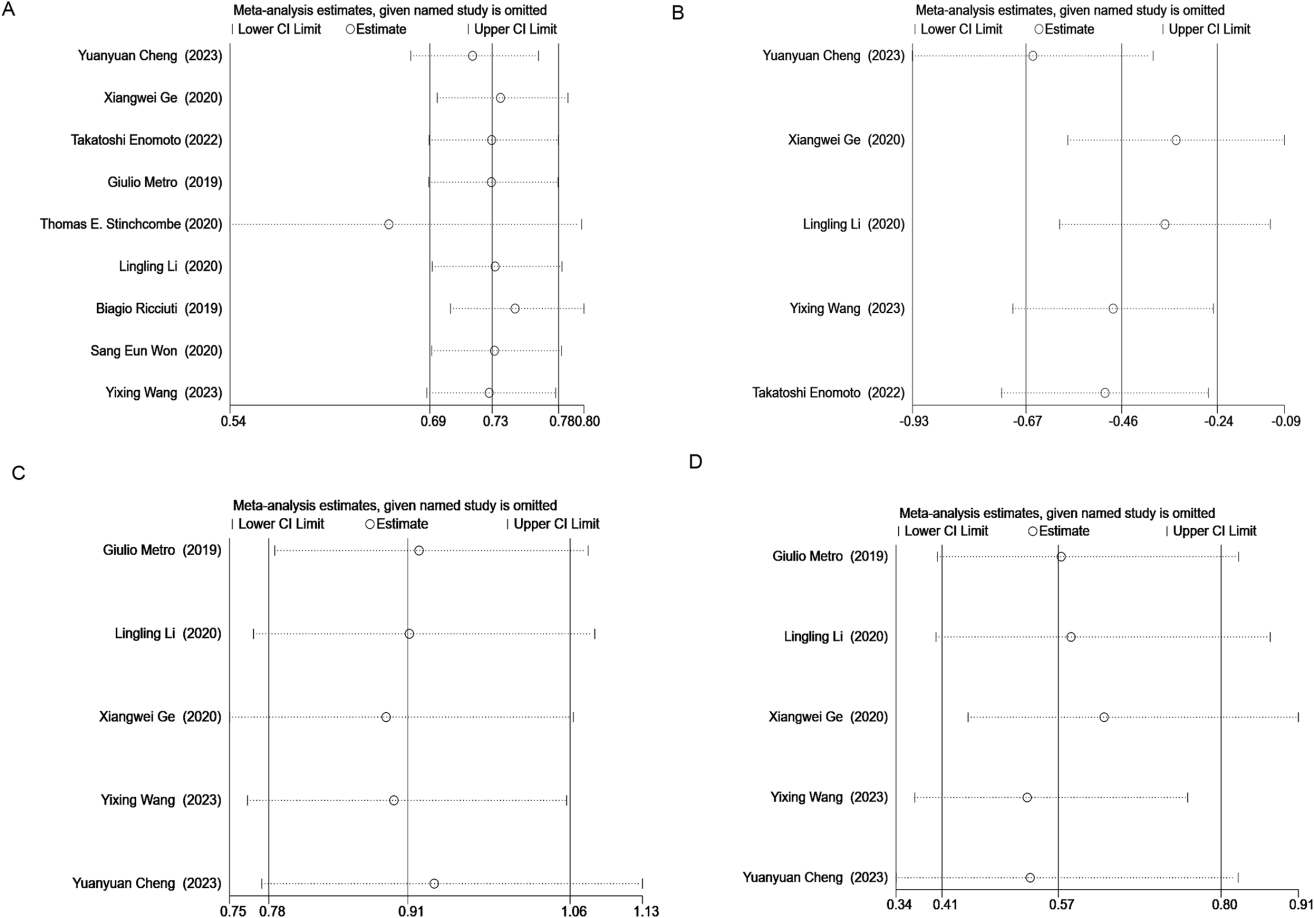
Results of sensitivity analysis for OS **(A)**, PFS **(B)**, ORR **(C)**, and DCR **(D)**.

Sensitivity analysis was performed on OS, PFS, ORR, and DCR using STATA 16.1. The results indicated that removing the study by Thomas E. Stingcombe (15) affected the stability of the OS data, but the PFS, ORR, and DCR results remained stable. Removal of each paired study did not significantly alter the meta-analysis outcomes (**Figure 8**).

Publication bias was assessed using Begg’s and Egger’s tests, with no significant evidence of publication bias (P > 0.05) for any outcome indicator (**Table 3**).

**Table 3:** Results of Begg’s test and Egger’s test.

Outcomes	Begg’s test	Egger’s test
OS	P = 0.602	P = 0.724
PFS	P = 1.000	P = 0.276
ORR	P = 1.000	P = 0.821
DCR	P = 1.000	P = 0.936

### 3.7 Safety

**Figure 9:**
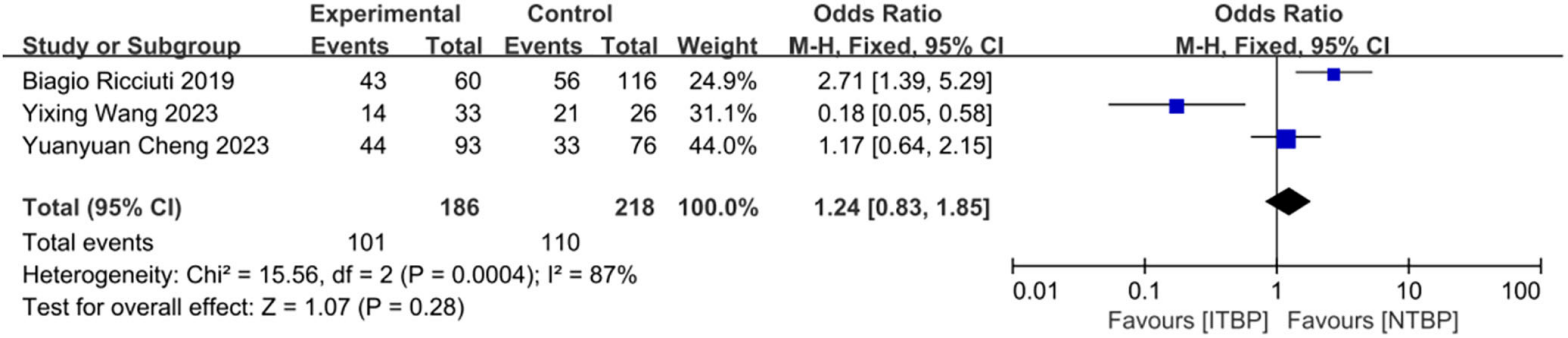
Forest plot for safety analysis of ITBP.

**Figure 10:**
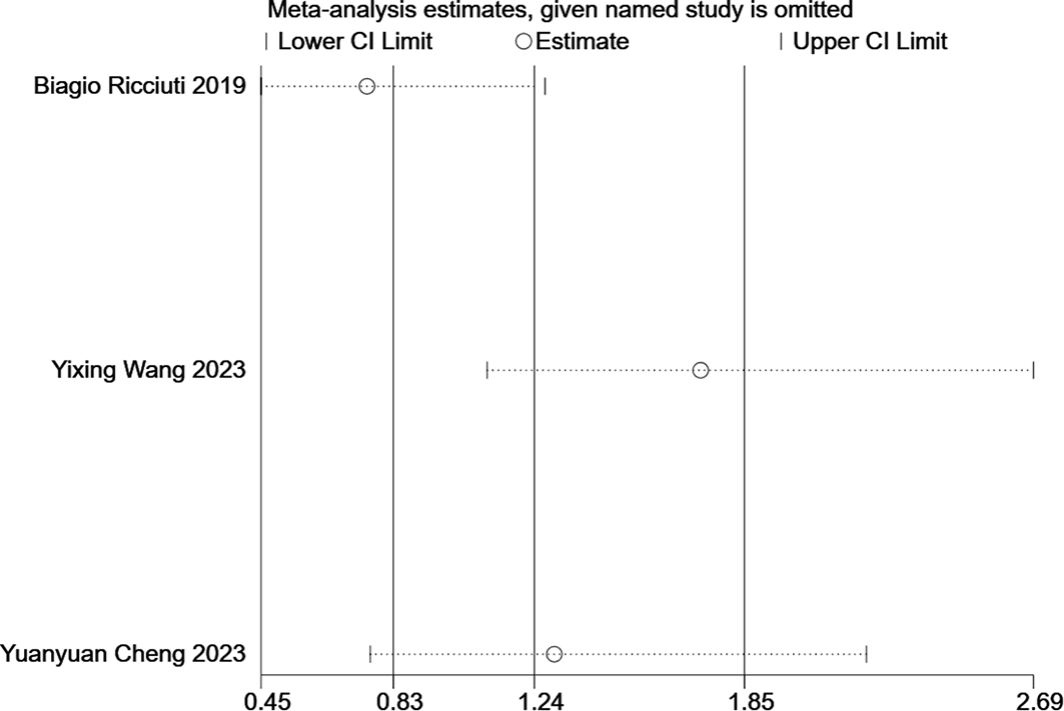
Results of sensitivity analysis for irAEs.

Three studies reported adverse events related to ITBP, including hematological (e.g., myelosuppression, anemia), gastrointestinal (e.g., nausea, vomiting), immune-related (e.g., pneumonia), skin (e.g., rash), and endocrine (e.g., thyroid dysfunction) reactions (**Table 4**, **Supplementary Figures S2**-**S10**). The meta-analysis found no significant difference in the overall incidence of adverse events between the ITBP and NTBP groups (OR 1.24, 95% CI 0.83-1.85, P > 0.05) (**Figure 9**). However, high heterogeneity was observed (I² = 87%), and subgroup analysis was not feasible due to the limited number of studies. Sensitivity analysis revealed that the study by Ricciuti (19) significantly altered the effect size when excluded, while the study by Cheng (17) had the opposite effect. This finding indicates that these two studies substantially influence the overall stability of the results and are likely contributors to the observed heterogeneity in adverse reactions (**Figure 10**). Furthermore, no significant differences were observed in the nine specific adverse reactions. However, nausea and vomiting, as well as myelosuppression, may represent potential adverse events associated with ITBP therapy and warrant further investigation and clinical vigilance.

**Table 4:** Meta-analysis results of the occurrence of irAEs after ITBP.

Adverse events	No. of included studies	No. of ITBP	Heterogeneity I^2^	OR	95%CI	P
Nausea and vomiting	2	93	74%	0.92	0.32-2.65	0.05
Fatigue	2	93	0%	2.24	1.11-5.39	0.44
Rash	2	153	30%	0.97	0.42-2.22	0.23
Anemia	2	93	0%	1.13	0.18-7.31	0.65
Abnormal liver function	2	153	29%	1.00	0.49-2.04	0.24
Thyroid insufficiency	2	153	0%	1.84	0.94-3.60	0.78
Adrenal insufficiency	2	153	0%	0.89	0.13-6.03	0.41
Myelosuppression	3	186	67%	0.73	0.41-1.30	0.05
Pneumonitis	2	153	1%	2.44	0.39-15.29	0.31

### 3.8 Best response of the initial immune response

**Figure 11:**
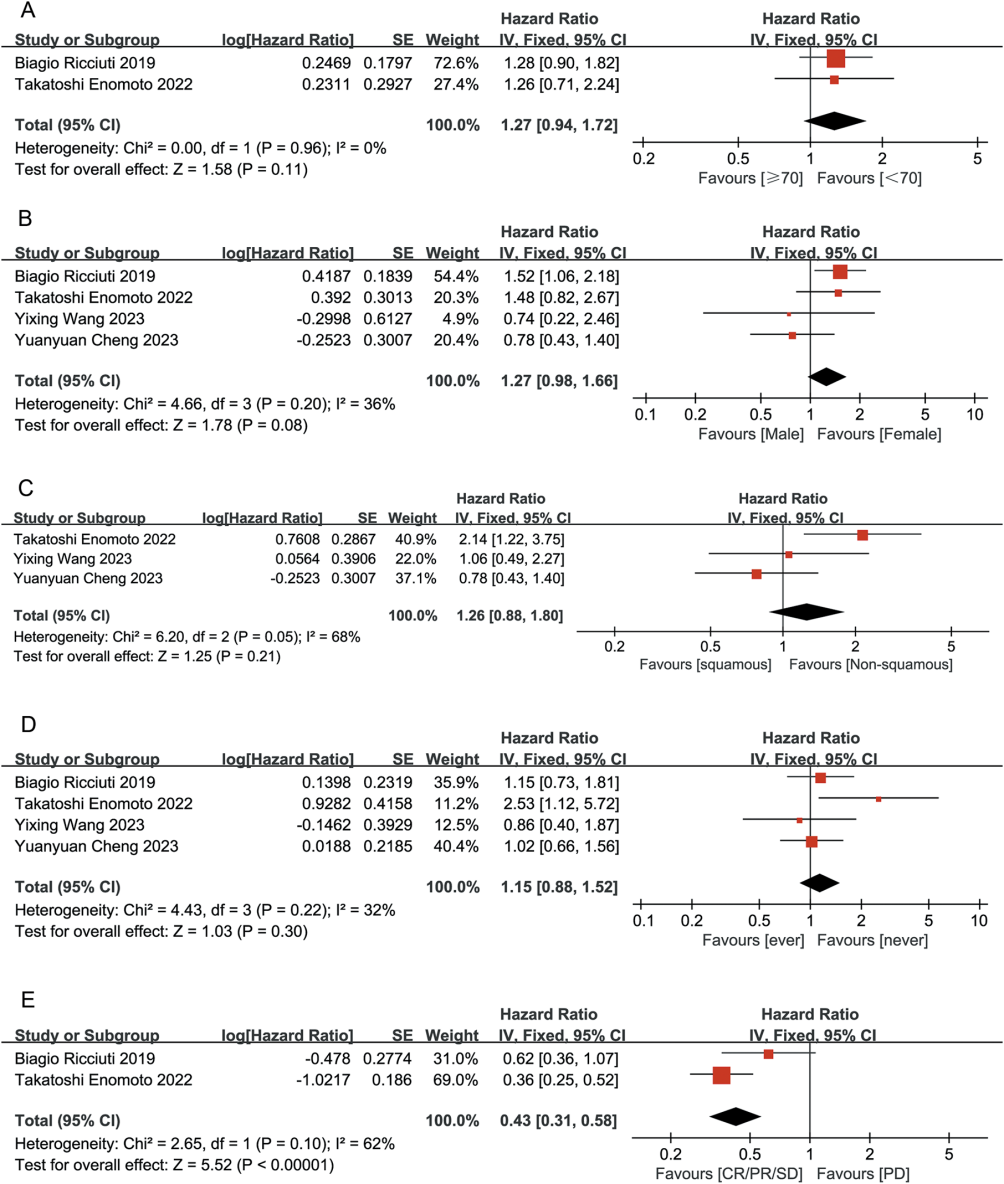
Forest plot summarizing the characteristic of population benefiting from ITBP for lung cancer. **(A)** Age; **(B)** Gender; **(C)** Lung cancer type; **(D)** Smoking history; **(E)** Initial immune response.

To optimize the benefit of ITBP in lung cancer, it is crucial to identify patient characteristics that may predict a better response. Our analysis revealed that patients who achieved CR/PR/SD had significantly longer OS following ITBP treatment than those who experienced PD after prior immunotherapy (HR 0.43, 95% CI 0.31-0.58, P < 0.05). There were no significant differences in OS based on age, gender, lung cancer type, or smoking history (age: P = 0.11; gender: P = 0.08; lung cancer type: P = 0.21; smoking history: P = 0.30; **Figure 11**).

## 4 Discussion

The primary strength of this study lies in its inclusion of RWS, which are typically more reflective of diverse patient populations, longer follow-up periods, and actual clinical practices, including patient treatment choices and processes. This approach enhances the ability of physicians to make treatment decisions that align with real-life circumstances. The findings from this study offer new insights into lung cancer treatment and suggest more precise therapeutic strategies that can significantly improve the quality of life for patients. The results demonstrate that, compared with NTBP, ITBP significantly prolonged both PFS and OS in patients with lung cancer. Additionally, ITBP did not result in a higher incidence of immune-related adverse events (irAEs). These findings strongly suggest that ITBP may be a viable therapeutic option for subsequent treatments in patients with lung cancer. Several factors underpin this conclusion. First, between 0.6% and 5.8% of patients with lung cancer undergoing immunotherapy may experience pseudo-progression. Accurate identification of pseudo-progression requires comprehensive evaluation, including clinical manifestations, imaging characteristics, and biomarkers (25–27). Solely relying on imaging results to determine true progression can lead to premature discontinuation of treatment, depriving patients of potential benefits from continued immunotherapy. Second, tumors may develop resistance to initial immunotherapy, with evolving resistance mechanisms over time. ITBP can address these changing mechanisms, improving treatment efficacy. Additionally, initial immunotherapy may alter the tumor microenvironment, enhancing the ability of subsequent ITBP to penetrate tumor tissue and optimize therapeutic outcomes (26). Third, resistance to immunotherapy may be linked to the loss of tumor antigen expression (25). In such cases, subsequent treatment lines that combine immunotherapy with other modalities (e.g., chemotherapy, radiotherapy, targeted therapy) may improve the immune microenvironment or tumor vasculature, continuing to exert antitumor effects (28). In summary, ITBP represents a promising option for subsequent treatment in patients with lung cancer. However, clinical decision-making should consider multiple factors, including the patient’s clinical status, prior treatment response, resistance mechanisms, tumor biomarkers, and immune profile. It is also crucial to account for individual characteristics, such as age, comorbidities, and quality of life, while integrating the latest clinical evidence to develop the optimal treatment strategy.

Regarding safety, the results indicate that ITBP does not significantly increase safety-related risks in patients with lung cancer. However, a trend toward a higher incidence of nausea, vomiting, and myelosuppression (P = 0.05) was observed, which could impact patient quality of life, treatment adherence, and therapeutic outcomes. Additionally, as the number of treatment lines increases, irAEs such as thyroid insufficiency, adrenal insufficiency, and pneumonia may emerge. Therefore, individualized treatment and prevention plans should be developed based on baseline characteristics and risk profiles for adverse reactions. Effective management of these adverse events will require multidisciplinary collaboration. Future research should focus on identifying predictive biomarkers and developing preventive strategies to optimize ITBP treatment.

Subgroup analyses were conducted to explore efficacy differences in ITBP based on factors such as initial response to immunotherapy, smoking history, gender, and the presence of brain and liver metastases. The results consistently showed that ITBP outperformed NTBP in terms of both OS and PFS, regardless of subgroup. When analyzing patients based on age, gender, lung cancer subtypes, and smoking history, no statistically significant differences in OS were observed. However, a trend emerged suggesting that female patients aged 70 years or younger, with nonsquamous cell carcinoma and no smoking history, may experience more favorable therapeutic outcomes. Additionally, although comparisons between second-line and third-line ITBP did not yield statistically significant results due to the limited number of included studies (2-3 studies), a promising trend emerged indicating that ITBP may offer superior efficacy over NTBP in both second- and third-line settings. Larger studies are needed to confirm these trends and provide more reliable conclusions. This Thus, when implementing ITBP, patient-specific characteristics should be considered to optimize treatment plan selection. This trend aligns with findings from other studies suggesting that patients who respond well to first-line immunotherapy (i.e., achieving CR, PR, or SD) are more likely to benefit from continued ITBP (29). Furthermore, due to the substantial heterogeneity (I^2^ = 96%) observed among the three studies (19, 21, 22) eligible for inclusion in the ECOG score meta-analysis, only descriptive analyses could be performed, precluding a formal meta-analysis. This high degree of heterogeneity may be due to the article published by Enomoto (22), which reported that ECOG scores do not significantly affect patients’ PFS (P > 0.05). Our findings indicated that ongoing treatment with nivolumab does not yield significant benefits for patients with advanced NSCLC.

A previous study demonstrated that elevated expression levels of PD-L1 are frequently correlated with a favorable response to ICIs. In specific cancer types, such as NSCLC and melanoma, patients with PD-L1–positive tumors typically exhibit higher ORR and prolonged survival times when undergoing immunotherapy (30). Our systematic review/meta-analysis included a multicenter study from Europe, which included patients with NSCLC with PD-L1 expression ≥ 50%, compared the outcomes of salvage chemotherapy versus pembrolizumab (with or without local ablation therapy) in patients with advanced NSCLC exhibiting high expression of PD-L1 who had progressed after first-line immunotherapy. The results indicated that for patients who initially responded to immune therapy, achieving either PR or SD, and having two or fewer progression sites, the addition of local ablative radiotherapy to the progression site can optimize the benefits of pembrolizumab following disease progression. However, the role of PD-L1 as a predictive biomarker is not limited to a single therapy. In the combination of ICIs and chemotherapy, high expression levels of PD-L1 do not always predict improvement in treatment efficacy. Indicating that chemotherapy may increase the immunogenicity of tumors, thereby reducing the predictive value of PD-L1. In the bargain, other factors in the tumor microenvironment, such as tumor mutational load (TMB) and the presence of tumor-infiltrating lymphocytes (TILs), may also be associated with response to immunotherapy (31). Won et al. (21) showed that patients with a significant increase in TMB (>200% compared with baseline) after the first PD evaluation according to RECIST 1.1 had less benefit from ITBP. Nevertheless, this study failed to integrate PD-L1 expression levels with TMB to ascertain the characteristics of a more probable beneficiary population. Hence, the expression level of PD-L1 may serve as a significant factor influencing ITBP, although it should not be regarded as the sole determinant. Future large-scale predictive studies that incorporate multiple biomarkers are essential to validate our current conclusions. In clinical practice, it is crucial to integrate various biomarkers alongside clinical information to facilitate optimal decision-making in treatment strategies.

This study has several limitations. First, the limited sample size of the included studies restricted the ability to perform meta-analyses for certain outcome measures and patient subgroups. Second, like most systematic reviews and meta-analyses, the analysis is constrained by the data reported by the authors of the original studies. For some studies that did not provide HRs, we had to estimate HRs and related statistics, which may introduce some degree of error. Third, the majority of the studies focused on NSCLC (eight studies), with only one study addressing SCLC, potentially introducing bias into the findings. Fourth, economic evaluations were not included due to the limitations of the original studies. To address these limitations, we are currently conducting a single-center observational RWS on ITBP for lung cancer. This ongoing study is collecting data on patient safety, efficacy, and cost-effectiveness, which will provide a more comprehensive evaluation of treatment outcomes.

## 5 Conclusion

In conclusion, this study confirms that ITBP is an effective treatment for lung cancer in specific patient populations, offering superior efficacy compared with traditional subsequent-line therapies without increasing the incidence of irAEs. Female patients with nonsquamous lung cancer, no smoking history, aged ≤70 years, and who achieved a best prior response of CR/PR/SD are more likely to benefit from ITBP. Despite certain limitations, the study provides valuable insights for clinicians. Future large-scale RWS are needed to validate these findings and explore the integration of other treatment modalities to optimize ITBP. Additionally, economic evaluations should be conducted to provide a comprehensive basis for clinical decision-making.

## Data Availability

The original contributions presented in the study are included in the article/**Supplementary Material**. Further inquiries can be directed to the corresponding authors.
